# Case report: *Coxiella burnetii* vascular infection and lymphoma in the Netherlands

**DOI:** 10.1007/s15010-017-1061-9

**Published:** 2017-08-24

**Authors:** Sonja E. van Roeden, Cléa Melenotte, Mirjam H. A. Hermans, Harm A. M. Sinnige, Peet T. G. A. Nooijen, Gilles Audoly, Andy I. M. Hoepelman, Jan Jelrik Oosterheert, Didier Raoult, Peter C. Wever

**Affiliations:** 1 Department of Internal Medicine and Infectious Diseases, University Medical Center Utrecht, Heidelberglaan 100, 3584 CX Utrecht, The Netherlands; 2 CNRS, IRD, INSERM, AP-HM, URMITE, IHU, Méditerranée Infection, Aix Marseille Université, 19-21 Boulevard Jean Moulin, 13385, Cedex 05, Marseille, France; 3 Department of Medical Microbiology and Infection Control, Jeroen Bosch Hospital, Henri Dunantstraat 1, 5223 GZ ’s-Hertogenbosch, The Netherlands; 4 Department of Internal Medicine, Jeroen Bosch Hospital, Henri Dunantstraat 1, 5223 GZ ’s-Hertogenbosch, The Netherlands; 5 Department of Pathology, Jeroen Bosch Hospital, Henri Dunantstraat 1, 5223 GZ ’s-Hertogenbosch, The Netherlands

**Keywords:** *Coxiella burnetii*, Q fever, Non-Hodgkin lymphoma

## Abstract

**Objectives and design:**

Non-Hodgkin lymphoma has been linked to infection with *Coxiella burnetii*, potentially through overproduction of IL-10 during infection with C. *burnetii*.

**Materials and methods:**

Description of a case report.

**Results:**

We describe a patient with retroperitoneal non-Hodgkin lymphoma and vascular infection with C. *burnetii*. Immunofluorescence staining and fluorescence in situ hybridization targeting specific C. *burnetii* 16S rRNA were performed on the retroperitoneal lymphoma tissue sample obtained at diagnosis of NHL. Both were strongly positive for the presence of C. *burnetii*.

**Conclusions:**

This case provokes questions regarding a potential association between C. *burnetii* and NHL, and underlines the importance of further exploration of this association.

## Case report

**Fig. 1:**
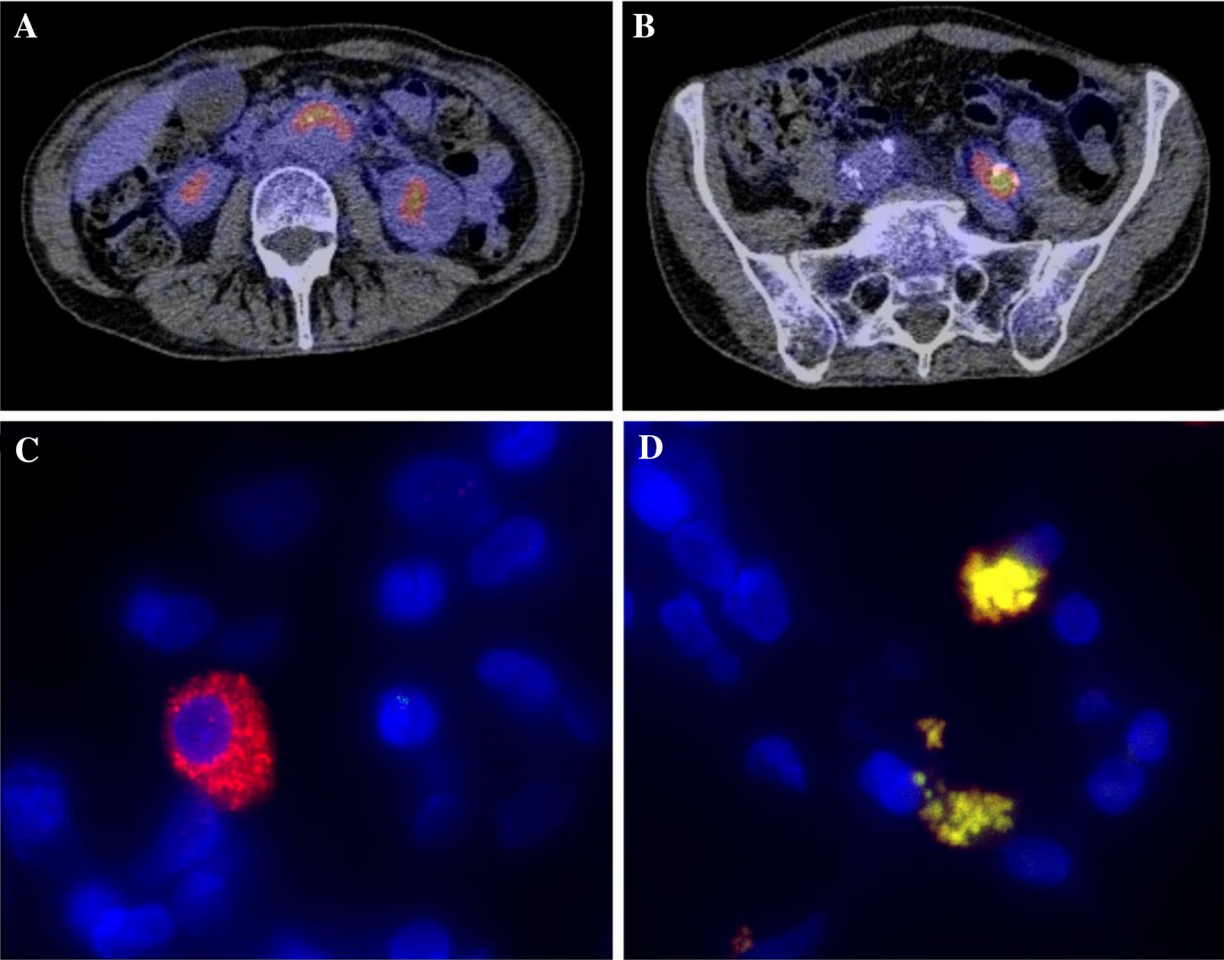
Composite figure of PET-CT, immunofluorescence (IF) and fluorescence in situ hybridization (FISH). **a** Highly increased ^18^F-FDG uptake in the aortic wall. **b** Highly increased ^18^F-FDG uptake in the left iliac artery. **c** Microscopic image (original magnification ×100) of immunofluorescence staining (IF) of retroperitoneal lymphoma tissue of a patient with vascular chronic Q fever in which nuclei are stained *blue* (4′,6-diamidino-2-phenylindole, DAPI), while perinuclear *Coxiella burnetii* is stained *red*. **d** Microscopic image (original magnification ×100) of fluorescence in situ hybridization (FISH) of the same tissue in which nuclei are stained *blue* (4′,6-diamidino-2-phenylindole, DAPI), while *C. burnetii*, organized in perinuclear vacuoles, is stained *yellow*. *Yellow* signal results of the co-localization of the universal probe EUB (*red*) and specific 16S rRNA *C. burnetii* probe (*green*). For both **c**, **d** Leica DMI6000 B microscope was used

A 58-year-old male patient, with a history of a vascular bypass because of occlusion of the infrarenal aorta in September 2010 and rheumatoid arthritis for which he used etanercept weekly, underwent abdominal ultrasound during routine follow-up in September 2012. He mentioned lower abdominal pain since a few weeks, but did not report any night sweats, fever, weight loss, rash or other complaints. He did not have any contact with animals, but lived in the Netherlands in an area where Q fever had been highly epidemic between 2007 and 2010 [1]. Laboratory results on presentation are shown in Table 1. On abdominal ultrasound, bilateral hydronephrosis with extensive retroperitoneal masses was observed. A subsequent computed tomography scan and positron emission tomography (PET) scan showed extensive retroperitoneal, intra-abdominal, mediastinal, cervical, and axillary lymphadenopathy, a pulmonary mass and a lesion in the right adrenal. In addition, the abdominal aorta and left iliac artery showed increased ^18^F-FDG uptake, which was not further analyzed. The PET-scan was made as part of the standard work-up procedure of suspected NHL, in accordance with Dutch guidelines [2, 3]. Biopsy of the retroperitoneal masses and a bone marrow biopsy revealed an Ann Arbor stage IV B cell non-Hodgkin lymphoma (NHL), with initially indefinite histopathological classification of the subtype of NHL. Re-examination of the pathology specimens at an academic hospital confirmed the presence of a B-cell NHL, with a marginal zone lymphoma as the most likely histopathological subtype. Chemo-immunotherapy (rituximab, cyclophosphamide, doxorubicin, vincristine, and prednisone) was initiated and etanercept was discontinued. After discontinuation of etanercept, the patient experienced two flares of his rheumatoid arthritis for which he received short courses of prednisone. Follow-up PET scan after three cycles of chemotherapy showed regression of all lymphoma locations, with the exception of pulmonary lesion, adrenal mass, and lower cervical lymph nodes. The increased ^18^F-FDG uptake in aorta and left iliac artery was unchanged. Because of unresponsiveness to chemotherapy of the pulmonary lesion, a second malignancy, possibly metastatic lung carcinoma, was suspected and further analysis was planned. Meanwhile, chemotherapy was discontinued, while awaiting analysis of the suspected lung lesion. Shortly after the last PET scan, the patient was diagnosed with pulmonary embolism for which heparin was started. Because of the use of anticoagulants and difficult accessibility of the pulmonary lesion, the diagnosis could not be confirmed pathologically. A follow-up PET scan was performed in February 2013, on which all NHL localizations were persistently in regression. However, the ^18^F-FDG uptake in the aorta and left iliac artery had strongly increased and the pulmonary lesion and pathological cervical lymph nodes were unchanged. The adrenal lesion was slightly larger. Patient was referred for second opinion and after due consideration did not want any further diagnostics and treatment of his pulmonary lesion. Analysis of the increased uptake in the aorta and left iliac artery was performed in June 2013. Repeated PET scanning revealed highly increased uptake of ^18^F-FDG of the abdominal aorta and left iliac artery, and a lesion suspicious of a small abscess near the left iliac artery (Fig. 1). As infection of the vascular bypass was suspected, blood cultures and serology for *Coxiella burnetii* were performed. Blood cultures were negative, but phase I and II IgG antibodies for *C. burnetii* were repeatedly positive with a maximum phase I IgG antibody titer of 1:4096 (Indirect Fluorescent-antibody Assay, Focus Diagnostics, Inc., Cypress, CA, USA). Phase I and II IgM antibodies against *C. burnetii* were negative. Polymerase chain reaction on serum was performed twice, but both samples tested negative. Vascular infection with *C. burnetii* was diagnosed according to both the Dutch chronic Q fever consensus group criteria and the criteria formulated by Eldin et al., and treatment with doxycycline (200 mg once daily) and hydroxychloroquine (200 mg three times daily) was started in July 2013 [4, 5]. No signs of endocarditis were present on physical examination or PET-CT, but an echocardiogram was not performed. In the absence of an echocardiogram, the presence of concomitant endocarditis could not be excluded with certainty [4, 6]. After start of the treatment, the patient experienced severe gastro-intestinal side effects and refused further therapy in September 2013. In March 2014, he reported repeated melena and rectal bleeding. An aortoduodenal fistula was suspected, but the patient did not want any further diagnostic interventions. His clinical condition deteriorated and he died the same month.

**Table 1:** Laboratory results at the day of presentation

Laboratory measurement	Result	Normal range	Units of measurement
Hemoglobin	7.2	8.5–11.0	mmol/L
Thrombocytes	179	150–400	10^9^/L
Leukocytes^a^	9.2	4.0–10.0	10^9^/L
Creatinine^a^	551	60–110	µmol/L
C-reactive protein	14	0–8	mg/L
Aspartate aminotransferase^a^	19	0–34	U/L
Alanine aminotransferase^a^	13	0–44	U/L
Gamma-glutamyltransferase^a^	<10	0–54	U/L
Alkaline phosphatase^a^	81	43–115	U/L
Lactate dehydrogenase^a^	246	0–247	U/L

^a^In the days following initial presentation, these laboratory values changed. Maximum values during admission were: leukocyte count 35.6 × 10^9^/L, C-reactive protein 186 mg/L, aspartate aminotransferase 35, alanine aminotransferase 49, alkaline phosphatase 177, gamma-glutamyltransferase 146, lactate dehydrogenase 400

We performed immunofluorescence staining (IF) and fluorescence in situ hybridization (FISH) targeting specific *C. burnetii* 16S rRNA on the retroperitoneal lymphoma tissue sample obtained at diagnosis of NHL in 2012, which were both strongly positive, Fig. 1. We refer to a previous article for technical details on IF and FISH for *C. burnetii* [7]. FISH is a relatively novel diagnostic method for detection of *C. burnetii,* with few studies using FISH targeting *C. burnetii* performed. The technique was applied in one clinical study and one case-report only [7, 8]. Melenotte et al. were able to detect *C. burnetii* in four out of seven lymphoma biopsies with FISH. All samples had positive immunofluorescence as confirmation, but negative immunohistochemistry [7]. Both FISH and IF are highly sensitive new diagnostic techniques to detect bacteria in situ, that may be superior to immunohistochemistry.

The presence of *C. burnetii* in the NHL tissue indicates that the infection was already present at the time of diagnosis of NHL in September 2012. At the moment of presentation in September 2012, our patient did not report any weight loss or fever. Nevertheless, he developed clear signs of inflammation shortly after admission (see Table 1). A potential explanation for the absence of fever at presentation may be the fact that our patient was immunocompromised. Absence of weight loss may be due to the fact that self-reporting of symptoms is not very accurate. In the Dutch national chronic Q fever database with data of 439 patients with persistent or chronic Q fever from the Netherlands, only 14% of patients had fever and only 28% of patients reported weight loss at presentation [9]. The absence of these symptoms illustrates that patients with a chronic infection can present with atypical symptoms.

It has been hypothesized that there is a causal relationship between persistent infection with *C. burnetii* and development of B-cell NHL [7]. Our patient presents similar as the index case of the series reporting a link between Q fever and B cell lymphoma: both patients had a vascular focus of infection and lymphoma located closely to the focus of infection [7]. A potential pathophysiological pathway is overproduction of IL-10 during infection with *C. burnetii*, which could play a role in the development of B-cell NHL [7, 10]. However, both diseases have a considerable diagnostic delay. It cannot be ruled out that *C. burnetii* infects monocytes and macrophages in tumorous tissue and that this patient had developed NHL before infection. Furthermore, both diseases have common risk factors, such as immunocompromised state [11, 12]. Our patient was severely immunocompromised, which is a risk factor for development of both the lymphoma and the vascular infection with *C. burnetii.*

In this report, we describe a case of NHL after Q fever. Naturally, no hard conclusions with regard to the association between *C. burnetii* and NHL and its causality can be drawn based on one single case. However, this case provokes further questions regarding the potential association between *C. burnetii* infection and NHL and its causality and potential diagnostic tools for detection of *C. burnetii* in tissues.

## References

[1] Dijkstra F, van der Hoek W, Wijers N (2012). The 2007–2010 Q fever epidemic in the Netherlands: characteristics of notified acute Q fever patients and the association with dairy goat farming. FMS Immunol Med Microbiol.

[2] Dutch society for Hematology (Nederlandse Vereniging voor Hematologie). Protocol on non-Hodgkin lymphoma. http://www.hematologienederland.nl/non-hodgkin-lymfomen. Accessed 24 July 2017.

[3] Hematology department University Medical Center Groningen. Protocol on non-Hodgkin lymphoma. https://hematologiegroningen.nl/protocollen/. Accessed 24 July 2017.

[4] Wegdam-Blans MC, Kampschreur LM, Delsing CE (2012). Chronic Q fever: review of the literature and a proposal of new diagnostic criteria. J Infect.

[5] Eldin C, Melenotte C, Mediannikov O, Ghigo E, Million M, Edouard S (2017). From Q fever to *Coxiella burnetii* infection: a paradigm change. Clin Microbiol Rev.

[6] Li JS, Sexton DJ, Mick N (2000). Proposed modifications to the Duke criteria for the diagnosis of infective endocarditis. Clin Infect Dis.

[7] Melenotte C, Million M, Audoly G (2016). B-cell non Hodgkin-lymphoma linked to Coxiella burnetii. Blood.

[8] Kumpf O, Dohmen P, Ertmer M (2016). Rapid molecular diagnosis of infective aortic valve endocarditis caused by *Coxiella burnetii*. Infection.

[9] Unpublished data from the national Dutch chronic Q fever database. Generated July 25th 2017.

[10] Levy Y, Brouet JC (1994). Interleukin-10 prevents spontaneous death of germinal center B cells by induction of the bcl-2 protein. J Clin Invest.

[11] Raoult D (1990). Host factors in the severity of Q fever. Ann N Y Acad Sci.

[12] Shankland KR, Armitage JO, Hanchock BW (2012). Non-Hodgkin lymphoma. Lancet.

